# Env mutations conferring improved entry efficiency allow HIV to replicate in the presence of IFN

**DOI:** 10.1186/1742-4690-10-S1-P93

**Published:** 2013-09-19

**Authors:** Sylvie Rato, Bénédicte Vanwalscappel, Fabrizio Mammano

**Affiliations:** 1 INSERM U941, Université Paris Diderot, Paris, France

## Background

Type-I interferons (IFN) inhibit HIV replication by inducing the expression of several cellular genes, some of which have direct antiviral activity. Forcing HIV to replicate in culture in the presence of IFN is expected to lead to the selection of variants with decreased susceptibility. The characterization of emerging viral variants may help in identifying the nature of the antiviral activities induced by IFN capable of affecting HIV replication.

## Methods

The R5-tropic HIV molecular clone NLAD8 was maintained in culture in a T-cell line (MT4R5) in the presence of IFNalpha2b (50 IU/ ml). At each passage, virus replication was compared in IFN-treated and untreated cells, until evidence of decreased susceptibility was observed. The genome of the selected virus population was then PCR-amplified and sequenced. Genomic segments carrying mutations were cloned into the parental NLAD8 provirus, to identify those that had an impact on IFN susceptibility. Single cycle and multiple cycle assays were performed to measure viral fitness and resistance to IFN.

## Results

HIV selection in culture led to the isolation of a variant with improved growth kinetics in the presence of IFN. This variant was characterized genotypically, and four mutations in the *env* gene (three in gp120 and one in gp41) were identified in 100% of the viral population. When transferred into the parental provirus, mutations in *env* accounted for the observed decrease in IFN susceptibility, and allowed virus replication in the presence of IFN concentrations that proved restrictive for the wild-type virus.

These mutations induced an increase of virus replicative capacity, detectable both in single cycle and in multiple cycle replication assays. Improved virus replication was associated with more efficient entry in the cytosol of target cells, as determined by a Vpr-BLam assay.

A similar pattern of V1 and V2 mutations in *env* was observed in an independent selection experiment in the presence of IFN. Interestingly, similar mutations in V2 were observed in one of two experiment of virus propagation in the absence of IFN. These V2 mutations conferred improved replication kinetics in this cell line, suggesting that the observed barrier to HIV replication was also present in untreated cells. When tested in the presence of IFN, the V2 mutant could also propagate significantly better than wild type.

## Conclusions

Virus propagation in tissue culture led to the emergence of variants characterized by improved virus entry. The higher infectivity of *env* mutants allowed them to replicate in the presence of IFN concentrations that prevented spread of the wild type virus. Evolution toward improved entry may represent an effective evolutionary strategy that allows the virus to overcome a variety of IFN-induced restriction factors.

